# Intranasal Immunization of Mice with Multiepitope Chimeric Vaccine Candidate Based on Conserved Autotransporters SigA, Pic and Sap, Confers Protection against *Shigella flexneri*

**DOI:** 10.3390/vaccines8040563

**Published:** 2020-10-01

**Authors:** Yrvin León, Lionel Zapata, Raúl E. Molina, Gaj Okanovič, Leonardo A. Gómez, Carla Daza-Castro, Manuel Flores-Concha, José L. Reyes, Angel A. Oñate

**Affiliations:** 1 Laboratory of Molecular Immunology, Department of Microbiology, Faculty of Biological Sciences, Universidad de Concepción, Concepción 4030000, Chile; yrvinln@gmail.com (Y.L.); ramolina@udec.cl (R.E.M.); leonardogomez@udec.cl (L.A.G.); carla.dazac@gmail.com (C.D.-C.); manuelflores@udec.cl (M.F.-C.); josereyes@udec.cl (J.L.R.); 2 Laboratory of Recombinant Biopharmaceuticals, Department of Pharmacology, Universidad de Concepción, Concepción 4030000, Chile; lionelzv@gmail.com; 3 Faculty of Medicine, University of Ljubljana, Ljubljana 1500, Slovenia; gaj.okanovic@gmail.com

**Keywords:** bacillary dysentery, *Shigella flexneri*, mucosal immunity, autotransporters, GroEL, multiepitope chimeric vaccine

## Abstract

Shigellosis is a diarrheal disease and the World Health Organization prompts the development of a vaccine against *Shigella flexneri*. The autotransporters SigA, Pic and Sap are conserved among *Shigell*a spp. We previously designed an in silico vaccine with immunodominat epitopes from those autotransporters, and the GroEL protein of *S.* typhi as an adjuvant. Here, we evaluated the immunogenicity and protective efficacy of the chimeric multiepitope protein, named rMESF, in mice against lethal infection with *S. flexneri*. rMESF was administered to mice alone through the intranasal (i.n.) route or accompanied with Complete Freund’s adjuvant (CFA) intradermically (i.d.), subcutaneously (s.c.), and intramuscular (i.m.), as well as with Imject alum (i.m.). All immunized mice increased IgG, IgG1, IgG2a, IgA and fecal IgA titers compared to PBS+CFA and PBS+alum control groups. Furthermore, i.n. immunization of mice with rMESF alone presented the highest titers of serum and fecal IgA. Cytokine levels (IFN-γ, TNF-α, IL-4, and IL-17) and lymphocyte proliferation increased in all experimental groups, with the highest lymphoproliferative response in i.n. mice immunized with rMESF alone, which presented 100% protection against *S. flexneri*. In summary, this vaccine vests protective immunity and highlights the importance of mucosal immunity activation for the elimination of *S. flexneri*.

## 1. Introduction

*Shigella* spp. are Gram-negative, non-motile, non-spore-forming, facultative anaerobic bacilli bacteria that invade the human colonic epithelium and cause bacillary dysentery or shigellosis [1]. This pathogen has a very low infectious dose of about 10–100 bacteria cells [1], mainly affecting children under five years of age [2], but also all age groups during outbreaks [3,4]. There are four serogroups of *Shigella*: *S. dysenteriae* (12 serotypes), *S. flexneri* (six serotypes), *S. boydii* (18 serotypes), and *S. sonnei* (one serotype), respectively [5]. The Global Burden of Disease estimates that *Shigella* is the second common cause of diarrheal deaths, with 164,300 annual deaths worldwide [6,7]. The main serogroup correlated with shigellosis in developing countries is *S. flexneri* [5]. The urgency of a vaccine against *Shigella* is needed by the increase of antibiotic resistance registered in many countries [8,9], prompting the World Health Organization to prioritize the development of a safe and effective vaccine against *S. flexneri* [2,10,11].

Most of the vaccine candidates evaluated against *Shigella* present a weak immune response [12], they and confer inadequate broad protection among the different serotypes of *Shigella* [13,14]. An approach for overcoming this current problem in *Shigella* vaccines is the use of conserved antigens between the different serotypes of *Shigella* strains, like Ipas proteins, which were used as vaccines in mouse models, or in silico analyses with the conserved proteins: SigA, Pic, and Sap [15,16].

SigA, Pic, and Sap are autotransporters encoded in the pathogenic island (PAI) SHI-1 of *S. flexneri*. SigA, and Pic are involved in the mechanisms of virulence and natural induction of IgG immunoglobulins in the host [17,18,19]. SigA causes severe damage to the Hep-2 cell line, and in volunteers infected with *S. flexneri* 2a, there are high levels of IgG antibodies against the protein [18]. Pic has mucinase activity and is considered the unique immune-modulating bacterial virulence factor in *Shigella*, comparing with a pic mutant that produces greater inflammation in the guinea pig keratoconjunctivitis infection model [19]. It is interesting to note that Pic from enteroaggregative *E. coli* (EAEC) induce high levels of IgG and IgM antibodies during a natural EAEC infection in children [20]. Sap, is a protein that has 87% identity with the amino acid sequence of Ag43 from *E. coli*, a protein that is involved in mechanisms of virulence, such as adhesion, aggregation, biofilm formation, and host immune evasion [17,21]. Hence, the described autotransporters could be considered to be targets in the development of vaccines against *Shigella* and some *E. coli* pathogenic specie [16].

Heat shock proteins (HSPs) are highly conserved proteins in bacteria and mammals [22]. These proteins interact with toll-like receptors (TLR), such as TLR2 and TLR4, and they stimulate the innate immune response [23]. Thus, HSPs, like GroEL (HSP 60) protein of *Salmonella* Typhi [24,25], have been successfully used as antigens [26] and adjuvants in the development of vaccine candidates [27]. The adjuvant capacity of GroEL has been evaluated in a recombinant domain-GroEL fusion protein with the conserved virulence protein IpaB of *Shigella* [28]. Mice immunized with the fusion protein as compared with mice without GroEL showed an increase in Th1 and Th2 response and high levels of protection in a *Shigella* lethal infection assay [28]. Moreover, the use of GroEL in fusion protein has the advantage of reducing the cost of vaccine production and simplifying manufacturing process and formulation [26,28,29].

Given this information and in consideration with a *S. flexneri* vaccine targeted at passenger domains of SigA, Pic and Sap autotransporter proteins, we implemented an in silico vaccine that was based on analysis of a chimeric multiepitope protein designed with highest immunogenic and antigenic epitopes bound to GroEL adjuvant [16]. In this work, we demonstrate that the immunization of mice with chimeric antigens displaying selected epitopes fused to GroEL induce an immune response and high protective efficacy against *S. flexneri*.

## 2. Materials and Methods

### 2.1. Mice

Six-week-old female BALB/c mice were provided by the Instituto de Salud Pública, Santiago, Chile. The animals were maintained in the Laboratory of Molecular Immunology, University of Concepcion, Chile. They were handled in all experiments while using standard conditions approved by the Bioethics and Safety Committee of the Faculty of Biological Sciences. Food and water were given ad libitum.

### 2.2. Growth Conditions of Bacterial Strains

*Shigella flexneri* 2a strain was kindly facilitated by Dr. Cecilia Toro of the Instituto de Ciencias Biomédicas, Universidad de Chile, Chile. *Shigella* was grown in tryptic soy broth (TSB, BD Difco™) at 37 °C. Colonies bearing the virulent plasmid pINV were selected on tryptic soy agar (TSA, EMD Millipore, Burlington, MA, USA) with 0.02% Congo red. *E. coli* DH5-α and *E. coli* BL21 strains used for cloning and expression, respectively, were grown in Luria Bertani (LB) medium (BD Difco™) at 37 °C with 50 µg/mL ampicillin where required.

### 2.3. Gene Construction and Cloning

We had previously designed an in silico gene construction with the sequences of the HSP GroEL of *S.* Typhi and the highly antigenic epitopes that were selected from three passenger domains of SigA, Pic, and Sap autotransporters of *S. flexneri*. These sequences were linked together with different proper linkers, EAAAK, GSGSAAY, and HEYGAEALERAG, according to the in silico design of vaccine that was described in León et al. [16]. The constructed gene was named multiepitope *S. flexneri* (MESF). It was optimized for *E. coli* expression and cloning into the vector pUC57 with two restriction sites *Sma*I and *Kpn*I added in the 3′ and 5′ ends, respectively (Biomatik Corporation, Wilmington, DE, USA). The construct was named pUC57-MESF. *E. coli* DH5-α was transformed with pUC57-MESF and the DNA plasmid was isolated with Wizard^®^ Plus SV Minipreps DNA Purification System as per manufacturer instructions (Promega, Madison, WI, USA). This was used to clone the MESF fragment into the vector pQE-80L (Addgene, Watertown, MA, USA) that has a 6xHis tag 5′ to the MCS site. The recombinant pQE-80L-MESF was electroporated into *E. coli* B21 and clones carrying the plasmid were screened using antibiotic selection.

### 2.4. Expression, Isolation and Purification of the Chimeric Fusion Multiepitope Protein

*E. coli* BL21 colonies bearing the pQE-80L-MESF were cultured in LB medium that was supplemented with ampicillin until OD_600_ of 0.6. Gene expression was induced with 1 mM isopropylthiogalactoside (IPTG) for 4 h at 37 °C, followed by centrifugation at 6000× *g* at 4 °C during 10 min. The pellet was suspended in lysis buffer containing Tris 50 mM, NaCl 200 mM, Imidazole 5 mM, pH 7.0, and maintained under 4 °C for 10 min. The cellular suspension was passed five times through high pressure condition while using a French press (Avestin, Ottawa, ON, Canada) with 8.2 × 10^7^ Pa under 4 °C. Subsequently, the suspension was centrifuged at 6000× *g* at 4 °C for 10 min. and the proteins in the soluble and insoluble fractions were evaluated and observed by sodium dodecyl sulphate polyacrylamide gel electrophoresis (SDS-PAGE) stained with Coomassie blue. The expressed fusion protein was confirmed through western blot by probing with IgG primary anti-Hisx6 tap and secondary anti-IgG HRP conjugated antibodies (Santa Cruz, Dallas, TX, USA). Once the expression of this protein was confirmed, the soluble fraction was analyzed by metal affinity chromatography via Ni-NTA column (ÄKTAprime, General Electric, Boston, MA, USA). The protein was eluted using elution buffer of Urea 8 M, Tris-HCl 20 mM, NaCl 200 mM, Imidazol 200–250 mM, pH 8.0. The eluted solution was dialyzed at 4 °C in buffer containing 50 mM Tris and 1 mM EDTA and concentrated (Amicon^®^ Ultra-4 Centrifugal Filter Units, Merck Millipore, Germany). The concentration of total protein was measured through the BCA method (Pierce™ BCA Protein Assay Kit, Thermo Fisher Scientific Inc., Waltham, MA, USA). The recombinant protein obtained (rMESF) was analyzed and confirmed by SDS-PAGE electrophoresis and western blot, as described above.

### 2.5. Immunization of Mice

**Figure 1:**
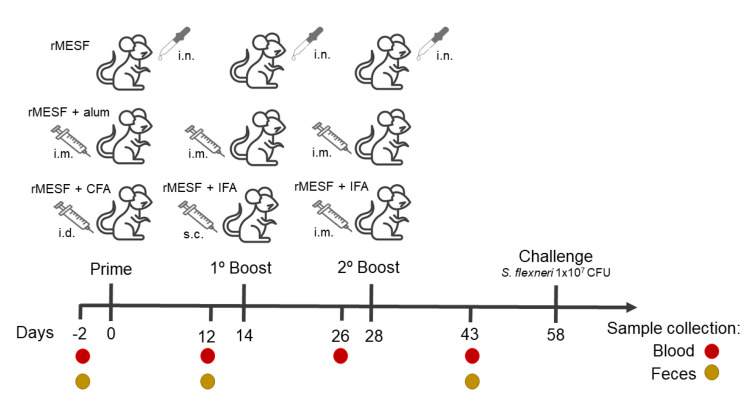
Immunization strategy and sample collection. Days of vaccine administration, adjuvants and routes are shown. The challenge assay was developed 30 days after the last immunization. Blood and feces collection are represented in red and golden brown circles, respectively. For more details of control groups read the text. rMESF, recombinant multiepitope *S. flexneri* protein. CFA, complete Freund’s adjuvant. IFA, incomplete Freund´s adjuvant. Alum, Imject^TM^ alum adjuvant. i.n., intranasal. i.m., intramuscular. s.c., subcutaneous.

Six-weeks old female BALB/c mice were randomly distributed into five groups (n = 10 per group) in different cages for the different routes of immunization. Group I was intranasally immunized (i.n.) with 25 µL of rMESF (25 µg/mouse, group rMESF), group II was injected intramuscularly (i.m.) with a mix of 50 µL of rMESF (25 µg/mouse) and 50 µL of Imject^TM^ alum adjuvant (Thermo Fisher Scientific, Waltham, MA, USA) (group rMESF+alum), group III was injected intradermally (i.d.) with a mix of 50 µl of recombinant protein (25 µg/mouse) and 50 µL complete Freund’s adjuvant (CFA, Sigma-Aldrich, St. Louis, MO, USA) (group rMESF+CFA). Subsequently two booster doses were given to all experimental mice groups on the 14th and 28th days after the first immunization. However, in the case of the group III, the subsequent two boosters were applied through subcutaneous (s.c.) and i.m. routes, respectively, with incomplete Freund’s adjuvant (IFA) on the 14th and 28th days. Two control mice groups were injected with phosphate Buffer saline (PBS) plus Imject^TM^ alum adjuvant (group IV, PBS+alum) and CFA (group V, PBS+CFA), respectively. A scheme is given with the immunization strategy that was used in this study (Figure 1).

### 2.6. Determination of Specific Antibodies in Peripheral Blood

Blood was drawn from the tail of each mouse from all five groups, two days before the first immunization, 12 days after the first and second immunizations, and 15 days after the last immunization, in order to evaluate the humoral immune response. The blood was centrifuged, and serum was isolated for specific IgG, IgG1, IgG2a, and IgA detection by Enzyme-Linked Immunosorbent Assay (ELISA). Briefly, 96-well plates were coated with 1 µg of the rMESF protein diluted in 200 µL of coating buffer (0.05 M carbonate-bicarbonate, pH 9.6). After overnight incubation at 4 °C, the plates were washed three times with PBS-0.05% Tween-20 (PBST) and blocked with PBS 0.8% gelatin for 2 h at 37 °C. Following blocking, the plates were washed with PBST three times, and each well was incubated with serial dilution of the serum from mice at 37 °C for 2 h. After washing three times with PBST, the plates were incubated for 1 h at room temperature with secondary antibodies rabbit anti-mouse IgG/IgG1/IgG2a or IgA (1:2000), conjugated to horseradish peroxidase (Serotec, Oxford, UK). Then 200 µL of the substrate TMB (3,3′,5,5′-Tetramethylbenzidine)/H_2_O_2_ (BD Biosciences, San Jose, CA, USA) was added and incubated in the dark for 30 min. at room temperature (RT), and the reaction was stopped by adding 50 µL H_2_SO_4_ 2N. The plates were read at OD_450_ nm using VictorX3 (PerkinElmer, Waltham, MA, USA). The antibody titers were expressed as mean ± standard deviation (SD) of log_10_ of the last reciprocal serum dilution above cut-off. The cut-off values were calculated, as described by Frey et al. [30].

### 2.7. Secretory IgA Determination

The level of secretory IgA (sIgA) was determined in the feces of immunized and control mice [31]. Feces were collected, weighed, homogenized, and diluted to 0.1 g/mL with PBS containing 0.1% sodium azide and 1 mM of phenylmethylsulfonyl fluoride (PMSF). Fecal suspension was centrifuged at 15,000× *g* for 5 min. at 4 °C, the supernatant fluid was recovered and again centrifuged at 15,000× *g* for 15 min. at 4 °C and stored at −80 °C until used. The specific IgA antibody levels were measured by ELISA with anti-IgA antibody (Thermo Fisher Scientific, Waltham, MA, USA), as previously described. The antibody titers were expressed as mean ± standard deviation (SD) of log_10_ of the last inverse serum dilution above cut-off. The cut-off values were calculated, as described by Frey et al. [30].

### 2.8. Lymphocyte Proliferation

Five mice per group were euthanized 30 days after the administration of the last immunization, and their spleens were removed under aseptic conditions. The spleens were used to prepare single-cell suspensions by mechanical disaggregation, and the erythrocytes were lysed using ammonium-chloride-potassium (ACK) buffer [32]. The cells were then washed with RPMI 1640 medium (Gibco^TM^, Life Technologies) and used to evaluate the lymphocyte proliferation in response to the chimeric multiepitope rMESF. Splenocytes suspended in RPMI medium that were supplemented with 10% heat-inactivated fetal bovine serum (Thermo Fisher Scientific, Waltham, MA, USA) and antibiotic/antimycotic solution (100 UI penicillin, 100 μg/mL streptomycin, and 0.25 μg/mL amphotericin B), were plated in 96-well flat-bottom plates (Nunc, Denmark), at a concentration of 1 × 10^5^ cells/well. The plates were sensitized with 1 µg/mL or 2 µg/mL of the chimeric protein and incubated for 72 h at 37 °C under 5% CO_2_. After this time, the cells were pulsed with 0.5 μCi tritiated thymidine (3H-TdR) per well (Amersham, Life Science, London, UK) and after 8 h DNA radioactivity was measure using a scintillation counter Beckman LS 6500 (Beckman, Indianapolis, IN, USA). The positive and negative controls consisted of 10 μg/mL of concanavalin A (Promega, Madison, WI, USA) and complete RPMI 1640, respectively. Cell proliferation data were expressed as the stimulation index of triplicate cultures from a cell pool from each group. These were obtained by dividing the amount of 3H-TdR incorporated (c.p.m.) in antigen-stimulated cell cultured divided by the c.p.m. derived from cells that were cultured without antigen [33].

### 2.9. Cytokine ELISAs

The levels of IL-4, TNF-α, IFN-γ, and IL-17 were measured by antigen-capture ELISA. Briefly, splenocytes were adjusted to a concentration of 4 × 10^6^ viable cells per ml in RPMI 1614 supplemented with 10% fetal calf serum (Thermo Fisher Scientific, Waltham, MA, USA) and antibiotic/antimycotic solution (100 UI penicillin, 100 μg/mL streptomycin, and 0.25 μg/mL amphotericin B). The cell suspensions were cultured in 24 well plates (Nunclon, Thermo Fisher Scientific, Waltham, MA, USA) and stimulated with the rMESF at a concentration of 1 µg/mL or 5 µg/mL or medium alone. They were incubated for 48 h at 37 °C under 5% CO_2_ to induce, in vitro, the expression of cytokines. After this time of incubation, the supernatants were collected and cytokines were quantified by ELISA sandwich using the mouse IL-4, TNF-α, IFN-γ, and IL-17 ELISA kits (BD Biosciences, USA), following the manufacturer instructions.

### 2.10. Challenge Studies

The protective efficacy of the candidate vaccine was evaluated in immunized groups (n = 5 mice/group) after 30 days of the last immunization. The lethal dose of *S. flexneri* 2457T (1 × 10^7^ colony forming units (CFU)/mouse) was determined by serial dilution, plating, and counting colonies [27,31]. Briefly, *S. flexineri* was grown in TSA with 0.05% Congo red at 37 °C, and then colonies were grown in TSB at 37 °C in agitation until an OD_600_ = 1. The bacterial culture was centrifuged at 6000× *g* for 10 min. and resuspended in PBS and adjusted to 5 × 10^5^ bacteria/µL for the infection. All of the groups of mice were challenged through the intranasal route with 20 µL, as described for the mouse pulmonary model with a lethal dose of 1 × 10^7^ CFU/mouse of *S. flexneri* [34]. The mice were observed for mortality for 30 days.

### 2.11. Organ Burden

Bacterial load in lungs was measured at three and 30 days after challenge assay in the control and experimental groups, respectively [28]. Three mice per group were euthanized, and the lungs were removed in aseptic condition. The lungs were homogenized in 5 mL of ice-cold PBS and they were 10-fold serially diluted. The bacterial serial dilutions were plated in LB agar at 37 °C, incubated for 18 h, and the CFUs were counted.

### 2.12. Statistical Analysis

Specific antibodies, cytokines levels, and lymphocyte proliferation were analyzed by two-way analysis of variance (ANOVA). The protection studies were expressed using Kaplan–Meier survival curves. Multiple comparisons were tested using Tukey Honest Difference. Graphs and statistical comparisons were generated by GraphPad Prism version 5.04 Software (GraphPad Software, San Diego, CA, USA). A *p* value of 0.05 or less was considered to be significant for all tests. All of the experiments were performed in triplicate.

## 3. Results

### 3.1. Gene Construction, Cloning, and Production of the Chimeric Recombinant Multiepitope Protein

**Figure 2:**
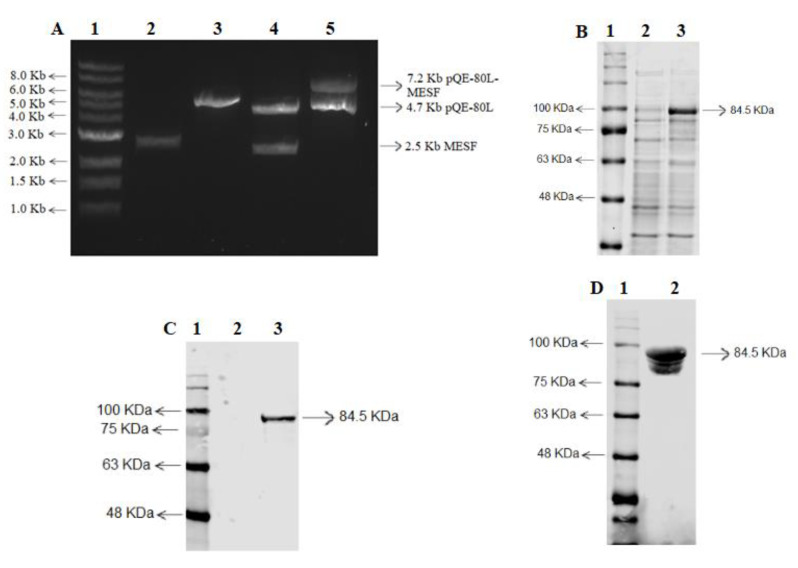
Cloning of chimeric gene (MESF), expression and purification of the recombinant multiepitope protein (rMESF). (**A**) Cloning of the MESF gene in pQE-80L expression vector with T4 ligase. Lane 1: 1 kb DNA ladder. Line 2: MESF gene. Line 3: pQE-80L. Line 4: product digestions of pQE-80L-rMESF with *Sma*I and *Kpn*I after ligation. Line 5: full circular size of pQE-80L-rMESP (**B**). SDS–PAGE of the expressed rMESF in *E. coli* BL21 cells after the induction with 1 mM IPTG for 4 h at 37 °C. Line 1: molecular mass marker. Line 2: protein before induction with IPTG. Line 3: proteins after induction with IPTG showing the rMESF protein with 84.5 kDa. (**C**) Western blot confirming the expression of rMESF after the induction with IPTG. (**D**) Western blot confirming the presence of rMESF protein after the purification-elution by Ni-NTA affinity chromatography. IPTG, isopropylthiogalactoside; SDS–PAGE, sodium dodecyl sulfate–polyacrylamide gel electrophoresis.

The gene for the recombinant multiepitope *S. flexneri* protein (rMESF) was obtained from Biomatik Corporation and then was cloned into the pQE-80L expression vector. Total size of rMESF was 2508 bp, the complete size of pQE-80L-rMESF was 7259 bp, and the digestion products with *Sma*I and *Kpn*I of the ligated vector (Figure 2A) were observed on agarose electrophoresis gel after extraction and purification from transformed *E. coli* BL21 (Figure 2A). The 6xHis tail is encoded in the expression vector at the 5’end of the gene. The recombinant protein was induced with 1 mM IPTG, and the rMESF protein expression was confirmed with a SDS-PAGE electrophoresis (Figure 2B) and western blot (Figure 2C). The expression of rMESF induced with IPTG showed that this recombinant protein has a molecular weight close to 84.5 kDa. The majority of this protein was found in the soluble fraction after membrane disruption of the bacteria. Elution and purification were carried out while using Ni-NTA chromatography. The refolded, dialyzed, and concentrated protein was confirmed by SDS-PAGE electrophoresis, followed by western blot (Figure 2D).

### 3.2. Chimeric Multiepitope Protein rMESF Induces Strong Humoral and Mucosal Immune Responses

**Figure 3:**
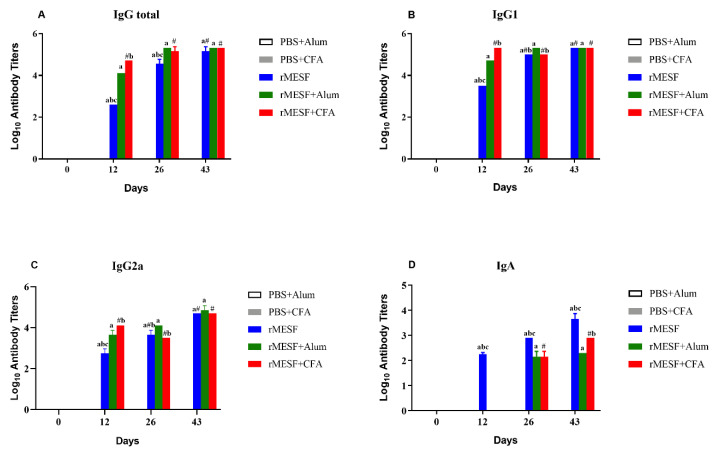
Antibody serum titers in controls and immunized mice. (**A**) Serum IgG. (**B**) IgG1. (**C**) IgG2a. (**D**) serum IgA. Controls, PBS+alum and PBS+CFA. rMESF+alum groups were immunized intramuscular (i.m.). rMESF +CFA were immunized through intradermic route (i.d.) and the first and second boosters were applied in this group through subcutaneous (s.c.) and i.m. routes, respectively with incomplete Freund’s adjuvant (IFA). rMESF groups were immunized with rMESF alone trough intranasal route (i.n.). The two booster doses were given to all experimental mice groups on the 14th and 28th days. Antibody titers were expressed as mean ± standard deviation (SD) of log_10_ of the last reciprocal serum dilution above cut-off. Symbols and letters indicate statistically significant values of *p* < 0.0001 compared with the respective mice group, # vs. PBS+CFA, a vs. PBS+alum. b vs. rMESF +alum, c vs. rMESF +CFA. PBS, phosphate-buffered saline. CFA, complete Freund’s adjuvant.

Specific antibodies for rMESF were measured in order to evaluate the humoral immune response. Overall, the immunoglobulins evaluated increase after the first immunization and were maintained over time, showing highly significant differences when compared with the controls PBS+CFA and PBS+alum (*p* < 0.0001). Serum from i.n. immunized mice with rMESF reached the highest levels of IgG and IgG1 after the second immunization (Figure 2B and Figure 3A). However, IgG (Figure 3A) and IgG1 (Figure 2B) in mice that were immunized with rMESF+CFA or rMESF+alum reached the highest levels after the first immunization, and there were no differences in both immunoglobulins between the three experimental groups after the third immunization. Furthermore, maximal peaks of IgG2a were reached by all of the experimental groups after the third immunization (Figure 3C), without significant differences between them.

**Figure 4:**
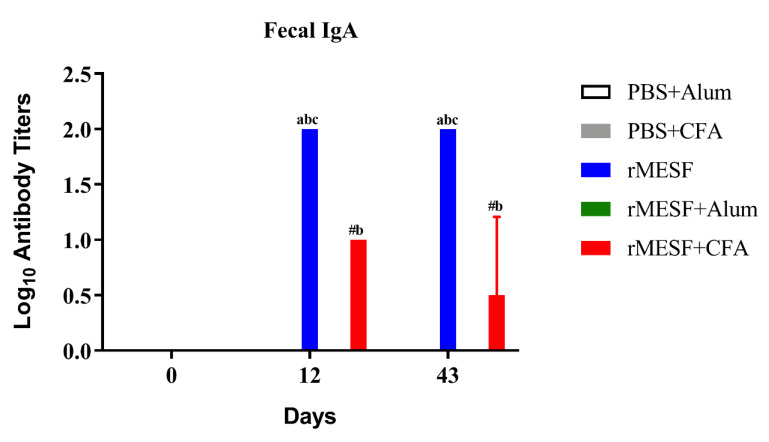
Antibody titers of fecal IgA in controls and immunized mice. Controls, PBS+alum and PBS+CFA. rMESF+alum groups were immunized intramuscular (i.m.). rMESF+CFA were immunized through intradermic route (i.d.) and the first and the second boosters were applied in this group through subcutaneous (s.c.) and i.m. routes, respectively with incomplete Freund’s adjuvant (IFA). rMESF groups were immunized with rMESF alone trough intranasal route (i.n.). The two booster doses were given to all experimental mice groups on the 14th and 28th days. Antibody titers were expressed as mean ± standard deviation (SD) of log_10_ of the last inverse serum dilution above cut-off. rMESF+alum group remains below the cut-off value calculated, Symbols and letters indicate statistically significant values of *p* < 0.0001 as compared with the respective mice group, # vs. PBS+CFA, a vs. PBS+alum, b vs. rMESF +alum, c vs. rMESF +CFA. PBS, phosphate-buffered saline. CFA, complete Freund’s adjuvant.

Serum IgA and fecal IgA displayed the highest levels in i.n. immunized mice with rMESF alone (Figure 3D and Figure 4, respectively) with highly significant differences (*p* < 0.0001) when compared with the control and rMESF+CFA or rMESF+alum groups. In general, all of the mice groups immunized presented predominant levels of IgG1 and IgG2a following IgA. Antibody titers in the serum and feces of the control animals remained below the limit of detection during the time of evaluation.

### 3.3. Multiepitope Protein Elicits a Cytokine Profile

**Figure 5:**
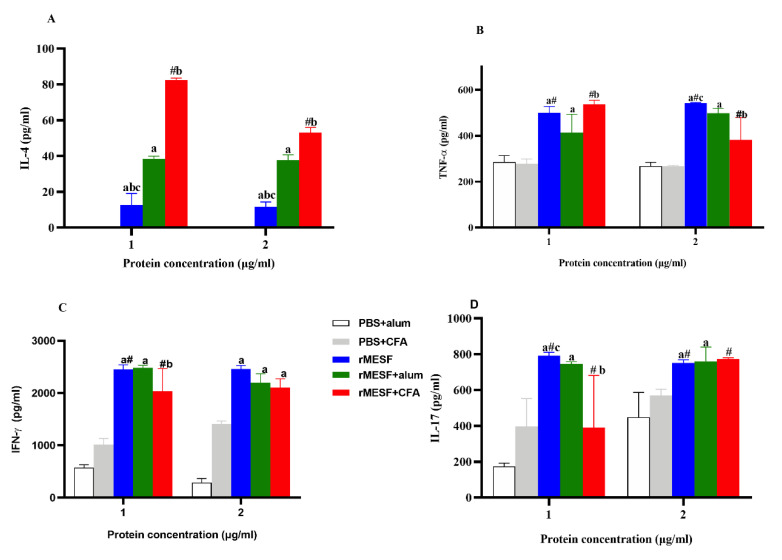
Cytokine levels quantified by ELISA sandwich. Production of (**A**) IL-4, (**B**) TNF-α, (**C**) IL-17 and (**D**) IFN-γ, present in the supernatant of splenocytes stimulated in vitro with 1 or 2 μg/mL of rMESF multiepitope fusion proteins for 72 h. Splenocytes were collected after 30 days of the last immunization (n = 5 mice/group). The absorbance was measured at 450 nm. Symbols and letters indicate statistically significant values with *p* values raging from <0.05 to 0.0001 (for more details read the text) as compared with the respective mice group. # vs. PBS+CFA, a vs. PBS+alum, b vs. rMESF+alum, c vs. rMESF+CFA. PBS, phosphate-buffered saline. CFA, complete Freund’s adjuvant. Results are plotted as mean ± standard deviation.

The cytokine profiles were evaluated from splenocytes cultured with the recombinant chimeric protein. The levels of IL-4 were significantly different (*p* < 0.0001) among all experimental groups and between them compared to the controls PBS+CFA and PBS+alum. However, lower levels of IL-4 were found in mice group i.n. immunized with rMESF alone, and the highest levels were presented in mice immunized with rMESF+alum and rMESF+CFA, respectively (Figure 5A). With respect to TNF-α, all of the experimental groups have significantly higher TNF-α levels when compared to the controls (*p* < 0.0001, Figure 5B).The highest levels of TNF-α were found in mice groups immunized with rMESF alone and rMESF+alum with significant difference (*p* = 0.002) between them and mice group immunized with rMESF+CFA. Regarding, IFN-γ and IL-17, these significantly increased in all experimental groups when compared with the controls, PBS+CFA and PBS+alum (*p* < 0.0001 for INF-γ and *p* = 0.03 for IL-17), with similar values between all experimental groups (Figure 5C,D).

### 3.4. Multiepitope Protein Elicits a Strong Lymphoproliferative Response

**Figure 6:**
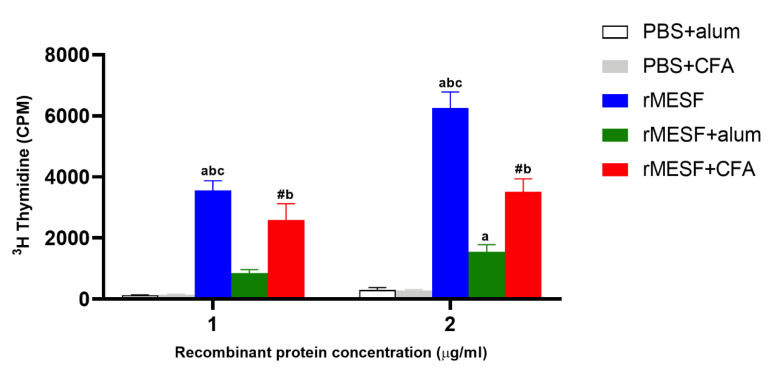
Lymphocyte proliferation. Splenocytes were collected from mice groups (n = 5 mice/group) after 30 days of the last immunization and stimulated in vitro with 1 μg/mL or 2 μg/mL with rMESF multiepitope fusion protein. Symbols and letters indicate statistically significant values of *p* < 0.0001 compared with the respective mice group. # vs. PBS+CFA, a vs. PBS+alum, b vs. rMESF + alum, c vs. rMESF + CFA. PBS, phosphate-buffered saline. CFA, complete Freund’s adjuvant. Results are plotted as mean ± standard deviation.

The lymphoproliferative response was evaluated on the splenocyte cells. Overall, the experimental immunized groups presented significant differences (*p* < 0.0001) in the lymphoproliferation against the multiepitope protein, compared with the respective controls, PBS+CFA and PBS+alum; there were also significant differences between the experimental groups. The highest levels of lymphoproliferation were found in mice immunized by i.n. route using rMESF alone, followed the mice groups immunized with rMESF+CFA and rMESF+alum adjuvant (*p* < 0.0001, Figure 6). All of the control groups did not display a lymphoproliferative response.

### 3.5. Multiepitope Protein Elicits Protective Response against Shigella Flexneri 2457T

**Figure 7:**
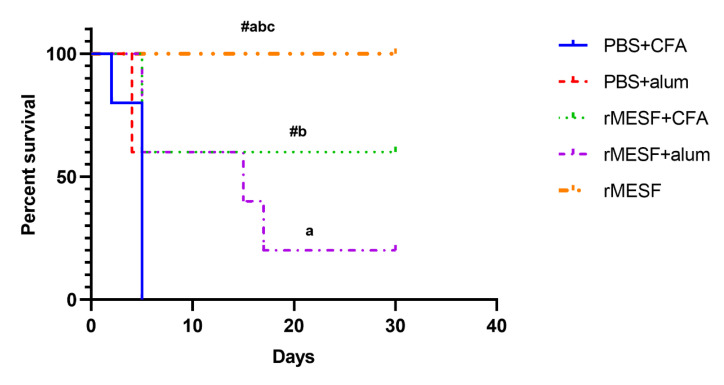
Protective efficacy of rMESF multiepitope fusion protein immunization on survival. Groups of female BALB/c mice (n = 5 mice/group) were immunized through different routes and adjuvant with 25 µg/mouse of rMESF. Thirty days after the last booster, mice were challenged with a *S. flexneri* lethal dose of 1 × 10^7^ CFU/mouse i.n. Mortality was observed for 30 days. Symbols and letters indicate statistically significant values of *p* < 0.0001 compared with the respective mice group. # vs. PBS+CFA, a vs. PBS+alum b vs. rMESF + alum, c vs. rMESF + CFA. PBS, phosphate-buffered saline. CFA, complete Freund’s adjuvant. i.n., intranasally. The protective effect was represented as Kaplan-Meier survival curves.

The strong humoral response and cytokines profiles were evaluated for a protective response against *S. flexneri*. The Kaplan–Meier survival curves showed that mice group i.m. immunized with rMESF+alum showed lower levels of protection, with 20% survival rate after the pulmonary model infection with *S. flexneri*. Moreover, the mice group immunized with rMESF+CFA showed 60% of survival after the infection. However, the highest survival percentage was observed in the group immunized with rMESF alone through i.n. route, where 100% of mice survived 30 days after the challenge assay (Figure 7). Control mice, PBS+CFA, and PBS+alum died within five days after the challenge assay.

### 3.6. Organ Burden

**Figure 8:**
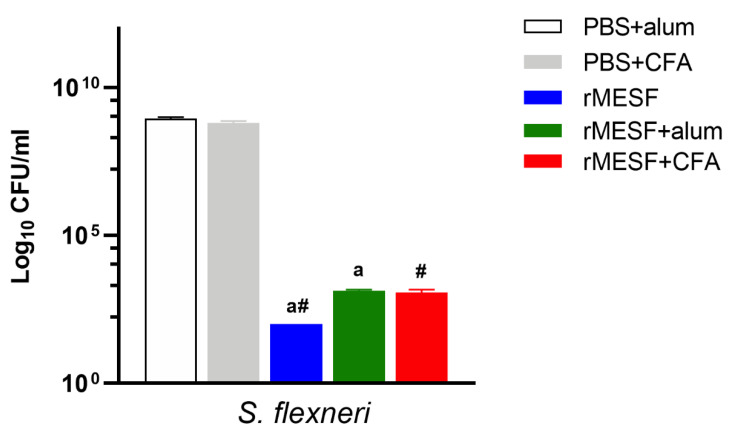
Bacterial burden in lungs after challenge assay of control and immunized mice. Lungs were collected from survival mice and controls groups after 30 days and three days, respectively, of challenge with lethal dose of *S. flexneri* (1 × 10^7^ CFU/mouse), and bacterial loads were determined by 10-fold serial dilutions on LB agar plates incubated at 37 °C for 18 h. Symbols and Letters indicate statistically significant values of *p* < 0.0001 compared with the respective mice group. # vs. PBS+CFA, a vs. PBS+alum. PBS, phosphate-buffered saline. CFA, complete Freund’s adjuvant. CFU, colony forming units; i.n., intranasally.

Organ burden studies were performed to determine the bacterial load in the lungs of control and surviving challenge mice in order to evaluate the ability of the chimeric multiepitope protein to promote the clearance of *S. flexineri*. The control groups showed the highest CFU in the lungs, while all of the surviving animals from the experimental groups showed a significant decrease (*p* < 0.0001) in bacterial load when compared to the controls, <1.3 × 10^2^ CFU/mL. Furthermore, mice that were immunized with rMESF alone through i.n. route showed the lowest bacterial load in lungs, <10^2^ CFU/mL, without significant differences between the surviving mice from rMESF+CFA and rMESF+alum groups (Figure 8).

## 4. Discussion

Bacillary dysentery is a global problem that affects children, immunocompromised patients, and elderly generally in developing countries. *S. flexneri* is one of the most important causing agents of shigellosis, and increasing bacterial antibiotic resistance prompts the development of safe and effective vaccines. Previously, we made an in silico design of a vaccine that contained an adjuvant, HSP-GroEL of *S.* Typhi, fused with highly immunodominant epitopes from the passenger domain of SigA, Pic, and Sap autotransporters proteins of *Shigella* [16]. The autotransporters that were chosen for the vaccine construction are well conserved in *Shigella* spp. and other *Enterobacteriaceae* that affect the human being [5,35,36].

A successful candidate vaccine against *Shigella* requires the induction of cell-mediated immunity, which plays an essential role in the elimination of these bacteria through the secretion of proinflammatory mediators from innate immune cells and increasing of CD8+ T cells-mediated cytotoxicity and cytokines secreted by CD4+ T cells inducing macrophages activation and the production of antibodies [27,31]. Moreover, the route of administration, the adjuvant and dose of vaccine are correlated with the vaccine effectiveness [29]. In this study, a robust humoral immune response induced during and after mice immunization by different routes, measured through IgG, IgG1, and IgG2a production, was strongly increased in all of the experimental groups. However, mice immunized through i.n. route showed a slower increase in the levels of immunoglobulins G compared with other routes. Many vaccines have been shown to confer high levels of protection in mice against *Shigella* by increased levels of IgG1 and IgG2a isotypes [27,28], as described for this candidate. Nevertheless, levels of systemic and mucosal IgA were increased in all experimental mice groups, with the exception of rMESF+alum mice group, but the highest levels were found in mice immunized through i.n. route. These results support the use of i.n. route for vaccination against *Shigella*, as it induces both systemic and mucosal immune protection [27,28,37]. In this context, systemic IgA is increased in patients infected with *Shigella* [38], while the levels of IgG and IgA are also increased in patients that naturally acquired immunity against *Shigella* [38,39]. Mucosal IgA is considered one of the most critical immunoglobulins in the clearance of *Shigella* and preventing reinfection [37]. Therefore, the induction of this antibody is key in the host protection, because IgA anti-LPS and other *Shigella* antigens interfere with bacterial invasion in the gut, and IgG facilitates the complement-mediated killing and phagocytosis of *Shigella* [37].

In *Shigella* infection, several cytokines and T cells population are activated in the host. IL-4, in conjunction with proinflammatory Th1 and Th2 cytokines, are extensively secreted in rectal biopsies from acute and convalescent adult patients with shigellosis that present severe mucosal inflammation [40]. In this work, the secretion of IL-4 varied significantly in all experimental groups, with the lowest levels in mice immunized through i.n. route. This is in accordance with results about the effect of adjuvants on immune response against *S.* Typhi, where IL-4 decreases when GroEL is used alone, compared when it is accompanied with CFA or alum adjuvants [29]. From this point, it could be interesting to know the exact levels of IL-4 that are necessary for an effective vaccine against *Shigella* and the central role of IL-4 in host protection. Additionally, cells produced high levels of IFN-γ in all experimental groups, which is a hallmark of *Shigella* infection [28,41,42,43]. IFN-γ induced by the vaccine indicates a robust Th1 lymphocyte response, which promotes the production of IgG2a by murine plasma cells [44]. This cytokine has been increasingly found in stool and plasma of convalescent patients compared with the acutely infected adults, which suggests IFN-γ plays an important role in the elimination of *Shigella* cells, being essential to develop an effective protective immunity against shigellosis [45].

Furthermore, the second-highest cytokine secreted was IL-17, which increased in all experimental groups. This cytokine is mainly produced by Th17 cells that are involved in pathogen clearance in mucosal tissues [46,47] and is considered a good indicator of vaccine protective efficacy [31]. In mice, an acute infection with *S. flexneri* induces a dominant Th17 response, playing an important role of secondary immune response that restricts bacterial growth after reinfection in this animal model [48]. Finally, the production of TNF-α, a cytokine associated to Th1 immunity was found to be increased in all experimental groups [49]. Nevertheless, the role of TNF-α in the immune response against *Shigella* is not clear, which is an open field of investigation in host protection against this pathogen.

Interestingly, in this study, the lymphoproliferative response was most potent in mice that were immunized through i.n. route with rMESF alone. This indicates the same recombinant vaccine has different effects depending on routes of vaccination and the adjuvant used. The lowest response was found in mice immunized i.m. with rMESF+alum, which is in accordance with other studies that reported a prominent induction of Th2 type by GroEL+alum with the lowest lymphoproliferative response [29,50]. The shift from Th2 to Th1 phenotype was supported with the highest lymphoproliferative response and levels of IFN-γ in mice that were immunized with rMESF alone or rMESF+CFA as compared to splenocytes isolated farom mice immunized with rMESF+alum group. Mechanisms of protection against *Shigella* are not clear, but activation of mucosal immune response is involved in the bacterial neutralization preventing the attachment to and the subsequent invasion of the host epithelial cells [27]. The immunization with HSP as an adjuvant in a fusion protein against HIV or *Shigella* elicit both humoral and cellular immune responses [28,51]. These observations demonstrate that vaccines designed with GroEL protein as adjuvant result in high levels of IgG1 and IgG2a or cytokines associated with the Th1 and Th2 immune response, and immunogenicity capacity of providing protection against *Shigella* [27,28].

Protection against intracellular pathogens requires a dominant Th1-type immunity [52]. This has been demonstrated with *S.* Typhi, an intracellular bacterium, which after immunization using alum adjuvant and GroEL, predominantly induced Th2 types response associated with the lowest protection levels [29]. Similar results were observed in this work, in mice that were immunized with rMESF+alum and rMESF+CFA, which secreted high levels of IL-4. Nevertheless, the low secretion of IL-4 should not be seen as a hallmark for protection, because mice immunized with rMESF+CFA secreted higher levels of IL-4 and better percentages of protection compared to mice immunized with rMESF+alum. Furthermore, i.n. immunization imparted 100% protection inducing the highest levels of systemic and secretory IgA, indicating a positive effect of this route in the protection against *Shigella*. Moreover, using GroEL as adjuvant avoids the several side effects that are caused by other adjuvants, such as excessive inflammation, arthritis, and other toxic effects of CFA observed in primates [29,53,54], and it has an advantage over alum adjuvant which only stimulates humoral immunity [55]. Besides, i.n. immunization in *Shigella* offers the opportunity to induce strong local and systemic protective immunity as it occurs during natural exposure [37]. The protection conferred by the vaccine was observed in the bacterial load in lung tissues, which revealed an enhanced elimination of *S. flexneri* cells, reducing the CFU in all surviving mice that were immunized through different routes compared with the controls. However, as expected, the lowest CFU were found in mice immunized i.n., <10^2^ CFU/mL. The decreased levels of CFU of *Shigella* in lung tissues of mice i.n. immunized is related to high levels of protection against *Shigella* spp. [27,28].

In conclusion, the present study showed that the immunization of mice with rMESF alone or in combination with adjuvants induced both humoral and cell-mediated immune responses. The percentage of protection offered by rMESF+alum (i.m.), rMESF+CFA (s.c.) and rMESFalone (i.n.) is 20, 60, and 100%, respectively. Differences in the protective efficacy were attributed to the nature of immune responses and the route of immunization. This is the first complete in vivo and challenge study using a mix of autotransporter proteins of *Shigella* fused to GroEL protein in a recombinant vaccine against *S. flexneri*. The results suggest the production of this recombinant vaccine without any adjuvant for preventing shigellosis caused by *S. flexneri* can be effective. However, the broad-spectrum protective efficacy of our vaccine should be evaluated due to the presence of SigA, Pic, and Sap proteins in *Shigella* spp. and others *E. coli* of photogenic importance.

## 5. Conclusions

The importance of using conserved antigens in *Shigella* such as SigA, Pic and Sap, and the intranasal vaccination and activation of the mucosal immune response are factors that influence the effective protective response against the pathogen. In our vaccine candidate, the protection is mainly related to the increase in immunoglobulin A, and using GroEL adjuvant as inducer of the immune response at the same time, we avoided the adverse effects caused by commercial adjuvants. This vaccine candidate represents an opportunity for the eradication of the major causal agent of shigellosis, *Shigella flexneri*, as well as a future hopeful candidate in cross protection against other subgroups of *Shigella* and pathogenic *E. coli*.
